# Laparoscopic versus open appendectomy for acute appendicitis in Sub-Saharan Africa

**DOI:** 10.1097/MS9.0000000000004445

**Published:** 2025-11-27

**Authors:** Temitomi Jane Oyedele, Syed Hasham Ali, Abigail Oghenefejiro Asini, Adepeju Anuoluwa Agunbiade, Komolafe Praise Dolapo, Idris Sandra Olufunmilayo, Adetola Emmanuel Babalola, Nicholas Aderinto

**Affiliations:** a Department of Medicine and Surgery, Bowen University, Iwo, Nigeria; b Department of Medicine, Dow University of Health Sciences, Karachi, Pakistan; c Department of Medicine and Surgery, Babcock University, Ogun, Nigeria; d Department of Medicine and Surgery, Afe Babalola University, Ado Ekiti, Nigeria; e Department of Medicine and Surgery, Kornberg School of Dentistry, Temple University, Philadelphia, USA; f Department of Medicine and Surgery, Ladoke Akintola University of Technology, Ogbomoso, Nigeria

**Keywords:** Africa, appendectomy, laparoscopy

## Abstract

**Background:**

Acute appendicitis remains a leading cause of emergency surgery globally. While laparoscopic appendectomy (LA) has become the preferred technique in high-income countries due to reduced postoperative complications and shorter recovery times, its adoption in Sub-Saharan Africa is limited by resource constraints. This systematic review and meta-analysis evaluates the safety, feasibility, and cost-effectiveness of LA compared to open appendectomy (OA) in this region.

**Methods:**

Following Preferred Reporting Items for Systematic Reviews and Meta-Analyses guidelines, a systematic search was conducted across PubMed/MEDLINE, Cochrane Library, AJOL, Scopus, Embase, and Web of Science from database inception to July 2025. Eligible studies included original research comparing LA and OA for acute appendicitis in Sub-Saharan Africa, with outcomes such as surgical site infection (SSI), operative time, hospital stay, and costs.

**Results:**

LA significantly reduced SSI risk [risk ratio 0.40, 95% confidence interval (CI): 0.23–0.69, *P* = 0.001, *I*^2^ = 0%] and hospital stay [mean defference (MD) 1.32 days, 95% CI: 1.64–0.99, *P* < 0.00001, *I*^2^ = 86%) compared to OA across 452 and 521 patients, respectively. Operative time was longer with LA (MD 13.79 minutes, 95% CI: 7.23–20.35, *P* < 0.0001, *I*^2^ = 78%). Cost data from two studies were inconclusive, with OA being less expensive in one (*P* < 0.001) and LA showing a borderline advantage in another (*P* = 0.049), with no significant difference in theatre costs (USD 634.92 versus USD 589.20, *P* = 0.264).

**Conclusions:**

LA offers significant safety and efficiency benefits over OA for acute appendicitis in Sub-Saharan Africa.

## Introduction

Acute appendicitis is the most common cause of acute surgical abdomen worldwide and remains one of the leading reasons for emergency surgery across all age groups^[1]^. The lifetime risk of developing appendicitis has been estimated at 7–8%, making it a significant global health problem^[2]^. Appendectomy, either through the conventional open technique or using laparoscopy, remains the gold standard for definitive management^[3]^. Over the past three decades, laparoscopic appendectomy (LA) has largely supplanted open appendectomy (OA) in high-income countries^[4]^. Numerous randomized controlled trials and meta-analyses conducted in Europe, North America, and Asia have consistently shown that LA offers several advantages over OA^[4,5]^. These include reduced postoperative pain, lower incidence of wound infections, shorter length of hospital stay, faster return to work and daily activities, and superior cosmetic outcomes^[5]^. The main disadvantages identified are longer operative times and higher direct health care costs, particularly related to disposable equipment and laparoscopic infrastructure^[4]^.

**HIGHLIGHTS**

- Laparoscopic appendectomy (LA) significantly reduced surgical site infections (by 60%) and shortened hospital stay by 1.3 days compared to open appendectomy.
- Although LA procedures took on average 13.8 minutes longer, cost comparisons were inconclusive across studies.
- The findings highlight the potential for wider adoption of laparoscopic surgery.

In contrast, the situation in low- and middle-income countries (LMICs), including Sub-Saharan Africa, is different. Despite the increasing burden of surgical diseases in the region, the uptake of minimally invasive surgery has been relatively slow^[6]^. Several barriers hinder its widespread adoption, including the high capital costs of laparoscopic towers, recurrent costs of consumables, limited training opportunities, and shortages of anesthetic and perioperative support^[6]^. In many district and rural hospitals, open surgery remains the default approach for acute appendicitis due to these constraints. However, as more tertiary centers in Sub-Saharan Africa acquire laparoscopic capacity and more surgeons are trained, LA is being performed in selected hospitals across Africa.

The emergence of LA in Sub-Saharan Africa raises important questions regarding its safety, feasibility, and cost-effectiveness in resource-constrained settings. From an economic perspective, the cost implications of laparoscopic versus OA in Sub-Saharan Africa remain debated. Despite the growing body of literature, the evidence remains fragmented. No comprehensive analysis of this evidence currently exists to provide clear guidance for clinicians, hospital administrators, and policymakers in Sub-Saharan Africa. Without such evidence, decisions on investment in laparoscopic infrastructure and training may be inconsistent, potentially widening inequities in access to high-quality surgical care across the region. This systematic review and meta-analysis seeks to fill this critical knowledge gap by synthesizing existing evidence on laparoscopic versus OA for acute appendicitis in Sub-Saharan Africa. This paper adheres to the TITAN guidelines^[7]^.

## Methodology

This systematic review and meta-analysis was conducted in accordance with the Preferred Reporting Items for Systematic Reviews and Meta-Analyses (PRISMA) guidelines^[8]^ and registered with PROSPERO. The study was reported in line with the AMSTAR (Assessing the methodological quality of systematic reviews) Guidelines.

### Eligibility criteria

Studies were considered eligible if they fulfilled the following criteria:

1. **Study design:** Original research articles comparing LA with OA. Both prospective and retrospective observational studies were eligible.
2. **Population:** Patients of any age presenting with acute appendicitis.
3. **Intervention and comparator:** LA (intervention) compared with OA (control).
4. **Outcomes:** At least one of the following had to be reported: population characteristics, perioperative outcomes (operative time, length of hospital stay), or clinical outcomes [surgical site infection (SSI)].
5. **Setting:** Studies conducted in Sub-Saharan Africa.

The following were excluded: case reports, case series, review articles, meta-analyses, narrative papers, conference abstracts, and studies involving non-human subjects.

### Literature search

A search was conducted in PubMed/MEDLINE, Cochrane Library, AJOL, Scopus, Embase, and Web of Science from database inception to July 2025. Reference lists of eligible articles were screened to identify additional studies. The search strategy used a combination of Medical Subject Headings (MeSH) terms and free-text keywords, including but not limited to: appendectomy, appendicitis, laparoscopic, open, and Sub-Saharan Africa. Search strings were tailored for each database and are presented in Supplemental Digital Content Table 1, available at: http://links.lww.com/MS9/B41. All retrieved citations were imported into EndNote for management, and duplicates were removed. Screening was performed at three levels: title, abstract, and full text. Two independent reviewers screened all articles. Disagreements were resolved by consensus or, when necessary, by a third reviewer.

### Outcomes of interest

For statistical robustness, only outcomes reported in three or more studies were considered for quantitative synthesis.

- **Primary clinical outcomes**:

- Length of procedure (minutes).
- Length of hospital stay (days).
- Incidence of SSI.

- **Secondary outcomes**:

- Direct financial data, including total hospital costs and theatre-only costs, when available.

All outcomes were extracted as reported in the original studies without modification.

### Data extraction

A predesigned Excel spreadsheet was used to extract data. The following information was collected from each study:

- Study characteristics (author, year, country, setting, and study design).
- Population demographics (sample size, age, and sex distribution).
- Intervention details (laparoscopic technique, number of ports, and surgeon experience).
- Comparator details (open technique specifics).
- Outcomes of interest (clinical, perioperative, and financial).

Two reviewers performed independent data extraction. Discrepancies were resolved by discussion.

### Risk of bias assessment

The Newcastle–Ottawa Scale was used to assess the quality of non-randomized studies. The tool evaluates three domains: selection of study groups, comparability, and ascertainment of outcomes. Independent assessments were carried out by two reviewers, and disagreements were resolved by consensus. Supplemental Digital Content Table 2, available at: http://links.lww.com/MS9/B42

### Statistical analysis

All statistical analyses were conducted using OpenMeta[Analyst] and Review Manager (RevMan) version 5.4.1 (The Nordic Cochrane Centre, Copenhagen, Denmark).

- **Dichotomous outcomes** (SSI): Results were expressed as risk ratios (RRs) with corresponding 95% confidence intervals (CIs).
- **Continuous outcomes** (operative time, length of stay): Results were reported as mean differences (MDs) with 95% CIs. When medians and interquartile ranges were reported, values were converted to means and standard deviations using the method of Wan *et al*.

A DerSimonian and Laird random-effects model was employed to account for heterogeneity in study design, outcome definitions, and surgical practice. Weighting for continuous outcomes was performed using inverse variance, while the Mantel–Haenszel method was used for dichotomous outcomes. Forest plots were generated for each outcome to illustrate pooled results. When heterogeneity exceeded 50%, sensitivity analyses were performed using a leave-one-out approach to evaluate the stability of results. Subgroup analyses and meta-regression were planned but not feasible due to the limited number of included studies. Formal assessment of publication bias was not conducted because of the small number of studies, which would render such analyses unreliable.

### Financial data standardization

Where financial outcomes were reported, costs were standardized to ensure comparability. Specifically:

1. All amounts were converted to United States Dollars (USD).
2. Values were adjusted for inflation to reflect July 2025 USD equivalents using the U.S. Department of Labor Statistics Consumer Price Index (CPI)^[9]^.

Because only a minority of studies reported detailed financial data, a narrative synthesis was performed in addition to limited pooled analysis.

## Results

### Literature search and study selection

**Figure 1.**
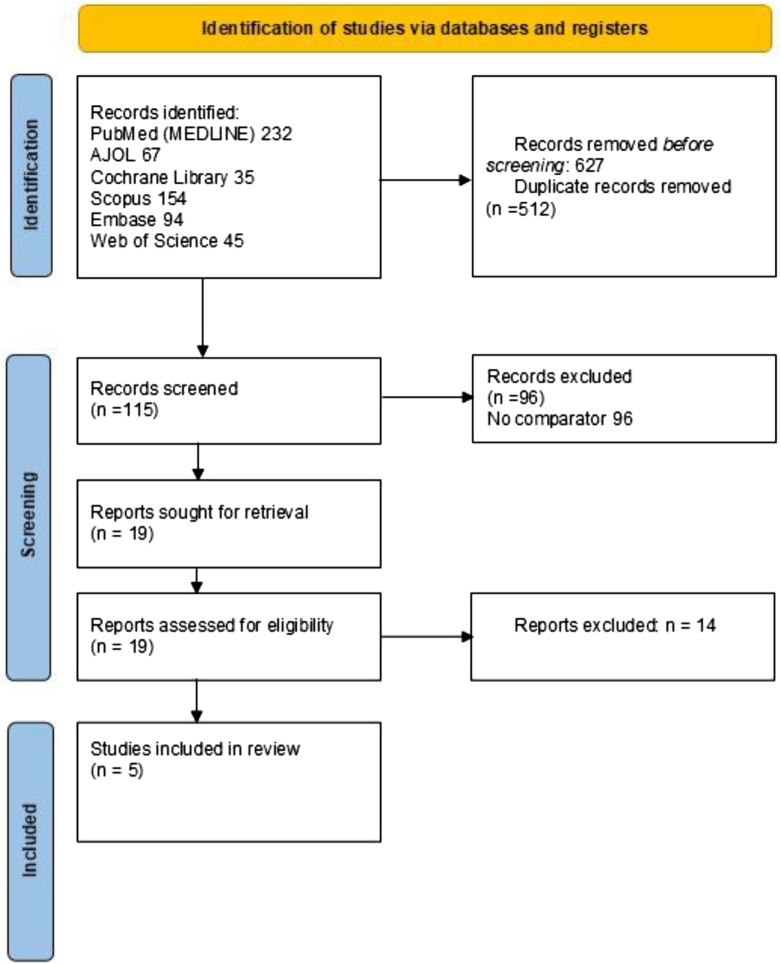
Screening process.

Our initial search yielded 627 records. Following removal of 512 duplicates, 115 records were screened, of which 96 were excluded for lacking a comparator. Full-text assessment of the remaining 19 reports led to the exclusion of 14 studies, resulting in five studies^[10–14]^ meeting the eligibility criteria for inclusion in the meta-analysis. The study selection process is summarized in the PRISMA flow diagram (Fig. 1).

### Study characteristics

All five included studies were retrospective in design and conducted at tertiary health care centers across Sub-Saharan Africa, with the majority originating from Nigeria^[5,6,8]^. Most studies spanned multiple years and reported broadly similar laparoscopic techniques, including comparable port placements^[12–14]^. Key features of each study, including sample size, study period, and surgical approach details, are summarized in Table 1.

**Table 1:** Study characteristics

*Study ID*	*Study design*	*Study centee*	*Study duration*	*Procedure- LA*	*Procedure- OA*	*Follow-up length*	*Outcome measures reported*
Adisa 2012 Nigeria	Retrospective	Ife Hospital Unit of the Obafemi Awolowo University Teaching Hospitals Complex, Ile-Ife, Osun State	January 2010—June 2011	Pneumoperitoneum was created in each patient by inserting a Veress needle at the umbilicus. An 11-mm trocar was then inserted at the same site for initial diagnostic laparoscopy. Before conducting an LA, a 5-mm port was introduced in the suprapubic region and another one in the right upper quadrant. The appendix was identified and freed from any adhesions by a combination of blunt and sharp dissection. The mesoappendix was then serially divided after coagulation with bipolar electrocautery, and the base of the appendix was secured using synthetic absorbable sutures by extracorporeal knotting. The specimen was extracted by drawing the appendix into the 11-mm port and extracting it under vision. There were few instances of markedly enlarged or purulent specimens, and a retrieval bag was used	Performed “conventionally.”	4 weeks	SSI, Length of Procedure, Length of Stay
Alegbeleye 2019 Nigeria	Retrospective	Laproscopic Surgery Unit, Lagoon Hospitals, Lagos	June 2013- December 2016	Before conducting an LA, a 5-mm port was introduced in the suprapubic region and another one in the left iliac fossa. The appendix was identified and freed from any adhesions by a combination of blunt and sharp dissection. “The mesoappendix was then serially divided after coagulation with bipolar electrocautery, and one the appendix base has been secured, and it is then divided between two PDS endo-loops (Roeder’s knots) or endo-clips.” The specimen was then retrieved in a retrieval bag through the 10 mm port under direct vision, and at times the improvised glove is used as an alternative to retrieval bag, which is equally cost-effective. The two 5 mm ports are then withdrawn after the peritoneal cavity has been inspected. The umbilical fascia was repaired with “O” PDS^®^ II (polydioxanone) suture on J needle and the skin with 3’O’ Vicryl^®^ (polyglactin 910; sub cuticular) suture, while the two 5 mm ports had only the skin approximated with 3’O’ Vicryl suture. The patient is discharged the next day, and follow-up period was between 2 and 3 months.	Performed “conventionally.”		SSI, Length of Procedure, Length of Stay
Gouws 2022 South Africa	Retrospective	Department of Surgery, New Somerset Hospital, Cape Town	1 January 2013- 31st December 2015				SSI, Length of Procedure, Length of Stay
Mba 2024 Nigeria	Retrospective	Federal Teaching Hospital, Gombe, Northeast	April 2018- September 2019	Done with the conventional three port system, pneumoperitoneum via Veress needle, the mesoappendix was diathermized, and an extracorporeal knot was applied at the base of the appendix	Done via Lanz incision; muscles were split, mesoappendix was ligated with Vicryl 0, and the base of appendix was transfixed and divided.	13 weeks	SSI, Length of Stay
Otoki 2025 Kenya	Retrospective	Tenwek Hospital, Bomet	1 January 2015 to 31 December 2019				SSI, Length of Procedure, Length of Stay

LA, laparoscopic appendectomy; OA, open appendectomy; SSI, surgical site infections.

### Baseline characteristics

Across the five studies, a total of 973 patients were included, comprising 452 patients in the LA group and 521 patients in the OA group^[10–14]^. The mean age of patients in most cohorts ranged from 20 to 30 years, reflecting a predominance of young adults presenting with acute appendicitis. Male patients were more commonly represented across all studies. Regarding surgical training and involvement, resident autonomy was exercised more frequently in OA procedures (255 cases) than in LA procedures (190 cases). Detailed baseline characteristics for each cohort are summarized in Table 2.

**Table 2:** Study characteristics

*Study ID*	*Adisa 2012*		*Alegbeleye 2019*		*Gouws 2022*		*Mba 2024*		*Otoki 2025*	
Arms	*LA*	*OA*	*LA*	*OA*	*LA*	*OA*	*LA*	*OA*	*LA*	*OA*
Population	31	83	145	113	225	168	20	20	31	137
Male/female	7/24	44/39	62/83	51/62	98/127	101/67	4/16	10/10	21/10	98/39
Age*	27 (15–51)	25.2 (14–68)	28.5 (15–55)	27.6 (14–65)	26 (19–32)	29 (21–37)	25.1 (7.5)	25.8 (5.6)	33 (28–45)	34 (26–44)
RLQ pain	31	31	145	113						
Nausea	20	56	130	104						
Vomiting	2	19	40	28						
Fever	4	13	22	18						
Guarding	6	39	28	14						
Ultrasound or CT scan findings	16	47	120	100						
Resident autonomy					178	154			12	101

LA, laparoscopic appendectomy; OA, open appendectomy; RLQ, right lower quadrant; SSI, surgical site infections.

^*^ Continuous data presented in mean (SD) or median (IQR).

### Meta-analysis outcomes

#### Surgical site infections

**Figure 2.**
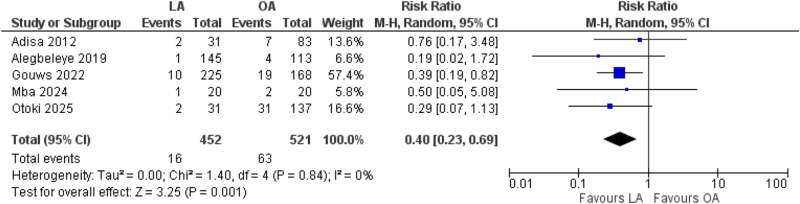
Forest plot of surgical site infections (SSIs).

All five studies reported data on SSIs. Pooled analysis demonstrated that the risk of SSI was significantly lower in the LA group compared with the OA group. Specifically, 16 of 452 patients in the LA group experienced SSI (3.5%), compared to 63 of 521 patients in the OA group (12.1%), corresponding to a risk ratio of 0.40 (95% CI: 0.23–0.69, *P* = 0.001, *I*^2^ = 0%)^[5–9]^ (Fig. 2).

#### Length of procedure

**Figure 3.**
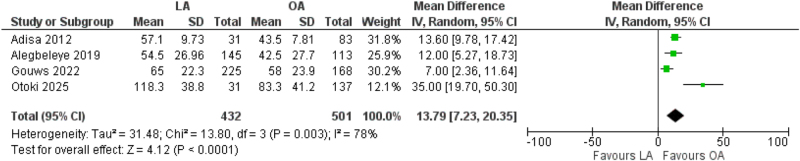
Forest plot of length of procedure.

Four studies reported operative time for both LA and OA procedures. Pooled results indicated that OA was associated with a shorter mean operative duration compared with LA, with a mean difference of 13.79 minutes (95% CI: 7.23–20.35, *P* < 0.0001, *I*^2^ = 78%)^[5,7–9]^ (Fig. 3). Total procedures analyzed included 501 OA cases and 432 LA cases. Heterogeneity was moderate to high; however, sensitivity analyses excluding the studies by Gouws *et al* and Otoki *et al* reduced heterogeneity to 0% without changing the direction or significance of the effect, suggesting a stable finding.

#### Length of hospital stay

**Figure 4.**
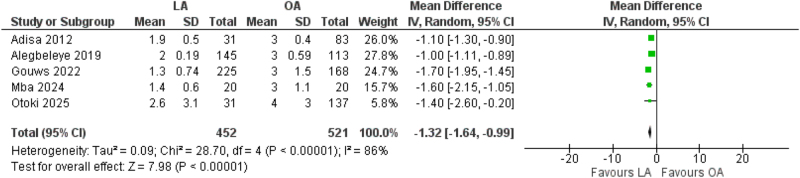
Forest plot of length of stay.

All five studies reported hospital length of stay. Meta-analysis revealed that patients undergoing LA experienced significantly shorter hospitalizations compared with those undergoing OA, with a mean reduction of 1.32 days (95% CI: 1.64–0.99, *P* < 0.00001, *I*^2^ = 86%)^[5,8,9]^ (Fig. 4). Sensitivity analyses indicated that the exclusion of Gouws *et al* reduced heterogeneity to 42%, while the effect size remained significant.

### Cost comparison

Two studies reported financial outcomes for LA and OA, with conflicting results^[11,14]^. Mba *et al* found that OA was significantly less expensive overall (*P* < 0.001), whereas Otoki *et al* suggested that LA may be less costly, though this result was only borderline significant (*P* = 0.049; Table 3). When considering only operating theatre costs, Otoki *et al* reported higher expenses for LA compared with OA (USD 634.92 versus 589.20), but the difference was not statistically significant (*P* = 0.264)*.

**Table 3:** Financial comparison

*Study ID*	*Direct cost- LA*	*Direct cost- OA*	*P-value*
Mba 2024	149.4 (1.78)	116.0 (3.25)	<0.001
Otoki 2025	1527.33 (1290.68–1763.97)	1815.89 (1683.78–1947.99)	0.049

* Values presented in mean (SD) or median (IQR).

## Discussion

This systematic review and meta-analysis offers an analysis of the evidence comparing LA and OA for acute appendicitis in Sub-Saharan Africa, with a focus on safety, feasibility, and cost outcomes. The significant reduction in SSI risk with LA is consistent with international studies conducted in higher-resource settings. Jaschinski *et al*^[15]^ across 67 randomized controlled trials reported a comparable SSI risk reduction (RR 0.43, 95% CI: 0.35–0.53), attributing this benefit to the minimally invasive nature of LA, which minimizes wound exposure. Similarly, the mean reduction in hospital stay by 1.32 days aligns with global findings^[16]^, though the elevated heterogeneity in this study reflects variability in postoperative care practices across Sub-Saharan African facilities. These parallels suggest that LA’s clinical advantages are transferable to resource-constrained environments, provided adequate infrastructure and expertise are available.

However, the longer operative time associated with LA, with a mean difference of 13.79 minutes compared to OA, parallels global observations. Dai *et al*^[17]^ reported a 15–20 minute increase in operative duration for LA in a meta-analysis of 45 studies, attributing this to the technical complexity and learning curve inherent to laparoscopic techniques. In the Sub-Saharan African context, this challenge is amplified by the limited number of trained laparoscopic surgeons and inconsistent access to modern equipment, as noted in the included studies. The high heterogeneity (*I*^2^ = 78%) in operative time data further suggests variability in surgeon experience and facility readiness, a finding supported by a 2022 study by Ng-Kamstra *et al* that highlighted the impact of surgical training disparities in LMICs^[18]^.

The cost comparison presents a more complex picture, diverging from global trends due to limited and inconsistent data. Only two studies reported financial outcomes, with Mba *et al* finding OA significantly less expensive (*P* < 0.001) and Otoki *et al* suggesting a borderline cost advantage for LA (*P* = 0.049). This contrasts with high-income country analyses, such as Kumar *et al*^[19]^, which reported higher direct costs for LA, driven by disposable equipment and infrastructure costs. The non-significant difference in theatre-only costs (USD 634.92 for LA versus USD 589.20 for OA, *P* = 0.264) in this review may reflect subsidized equipment in some Sub-Saharan African settings or variations in cost-reporting methodologies. A 2021 study by Wilkinson *et al*^[20]^ in LMIC contexts emphasized that societal cost savings such as faster return to work and reduced indirect costs often offset initial LA expenses. The standardization of costs to July 2025 USD equivalents using the U.S. CPI provided some comparability, yet the reliance on narrative synthesis highlights the urgent need for standardized economic evaluations in the region.

These findings carry significant implications for health care policy and practice in Sub-Saharan Africa. The demonstrated safety advantages of LA, coupled with its potential to reduce hospital stay, support its gradual integration into tertiary centers with sufficient infrastructure and trained personnel. However, the longer operative times and uncertain cost-effectiveness necessitate a phased approach, prioritizing capacity building through regional training initiatives and public-private partnerships to subsidize equipment costs, as suggested by Kebede *et al* (2023)^[21]^. The reliance on resident autonomy in OA (255 cases) versus LA (190 cases) also highlights a training gap that could be addressed through structured laparoscopic programs. Future research should prioritize prospective, multi-center trials with standardized outcome measures and comprehensive cost analyses, including indirect costs such as lost productivity, to provide clearer guidance.

## Limitations

All five included studies were retrospective, introducing potential selection and reporting biases. Also, the predominance of Nigerian studies (three out of five) limits the generalizability of findings across the diverse Sub-Saharan African region, where health care systems range from urban tertiary centers to rural district hospitals. The absence of heterogeneity in SSI results (*I*^2^ = 0%) and the stability of findings in sensitivity analyses lend credence to the clinical benefits, yet the high heterogeneity in hospital stay (I^2^ = 86%) and operative time data underscores the influence of local health care variability.

## Conclusion

This systematic review and meta-analysis of five retrospective studies encompassing 973 patients demonstrates that LA offers significant clinical advantages over OA for acute appendicitis in Sub-Saharan Africa. LA was associated with a 60% reduction in SSI risk and a shorter hospital stay by approximately 1.32 days, consistent with global evidence on the benefits of minimally invasive surgery. However, LA was also associated with longer operative times (MD 13.79 minutes), reflecting challenges related to surgical training and infrastructure, similar to observations from other LMICs.

## Data Availability

Data sharing is not applicable to this article as no datasets were generated.

## Research registration unique identifying number (UIN)

PROSPERO [CRD420251109301]. crd.york.ac.uk/PROSPERO/view/CRD420251109301.

## References

[1] Guan L Liu Z Pan G. The global, regional, and national burden of appendicitis in 204 countries and territories, 1990-2019: a systematic analysis from the global burden of disease study 2019. BMC Gastroenterol 2023 23:44. 36814190 10.1186/s12876-023-02678-7 PMC9945388

[2] Lotfollahzadeh S Lopez RA Deppen JG. Appendicitis. In: StatPearls [Internet]. Treasure Island (FL): StatPearls Publishing; 2025. Accessed 2024 Feb 12. https://www.ncbi.nlm.nih.gov/books/NBK493193/

[3] Schildberg C Weber U König V. Laparoscopic appendectomy as the gold standard: what role remains for open surgery, conversion, and disease severity? World J Emerg Surg 2025;20:11–13. 10.1186/s13017-025-00626-2 PMC12178000 40533760

[4] Biondi A Di Stefano C Ferrara F. Laparoscopic versus open appendectomy: a retrospective cohort study assessing outcomes and cost-effectiveness. World J Emerg Surg 2016;11:44. 27582784 10.1186/s13017-016-0102-5 PMC5006397

[5] Shiihara M Sudo Y Matsushita N. Therapeutic strategy for acute appendicitis based on laparoscopic surgery. BMC Surg 2023;23:161. 37312100 10.1186/s12893-023-02070-y PMC10265908

[6] Aderinto N Olatunji G Kokori E. Expanding surgical access in Africa through improved health insurance schemes: a review. Medicine (Baltimore) 2024;103:e37488. 38489736 10.1097/MD.0000000000037488 PMC10939550

[7] Agha RA Mathew G Rashid R. TITAN Group. Transparency In The reporting of Artificial INtelligence the TITAN guideline. Prem J Sci 2025;10:100082.

[8] Page MJ McKenzie JE Bossuyt PM. The PRISMA 2020 statement: an updated guideline for reporting systematic reviews. BMJ 2021;372:n71. 33782057 10.1136/bmj.n71 PMC8005924

[9] CPI Inflation Calculator n.d. accessed August 23, 2025. https://www.bls.gov/data/inflation_calculator.htm

[10] Adisa AO Alatise OI Arowolo OA. Laparoscopic appendectomy in a Nigerian teaching hospital. Jsls 2012;16:576–80. 23484567 10.4293/108680812X13462882737131 PMC3558895

[11] Mba EL Guduf MI Mshelia NM. Open versus laparoscopic appendectomy: a comparison of outcome in a teaching hospital, Northeast Nigeria. J West Afr Coll Surg 2024;14:392–95. 39309381 10.4103/jwas.jwas_165_23 PMC11412582

[12] Juan G Nazmie K Heather B. Laparoscopic appendectomy by surgical trainees at a public teaching hospital in Cape Town, South Africa: a retrospective, observational study. East Cent Afr J Surg 2022;3:79–85.

[13] Alegbeleye B Coker A Ohene J. LAPAROSCOPIC APPENDECTOMY IN LAGOON HOSPITALS. NIGERIA 2019;10:1231–36.

[14] Otoki K Simel I Moenga D. Laparoscopic appendectomy improves outcomes and reduces costs in rural Kenya. Surg Endosc 2025;39:2191–97. 39915311 10.1007/s00464-025-11589-5

[15] Jaschinski T Mosch CG Eikermann M. Laparoscopic versus open surgery for suspected appendicitis. Cochrane Database Syst Rev 2018;11:CD001546. 30484855 10.1002/14651858.CD001546.pub4 PMC6517145

[16] Procope B. Laparoscopic Versus Open Surgery For Suspected Appendicitis. Gastroenterol Nurs 2020;43:200–02. 32251221 10.1097/SGA.0000000000000508

[17] Dai L Shuai J. Laparoscopic versus open appendectomy in adults and children: a meta-analysis of randomized controlled trials. United European Gastroenterol J 2017;5:542–53. 10.1177/2050640616661931 PMC5446136 28588886

[18] Ng-Kamstra JS Arya S Greenberg SLM. Perioperative mortality rates in low-income and middle-income countries: a systematic review and meta-analysis. BMJ Glob Health 2018;3:e000810. 10.1136/bmjgh-2018-000810 PMC6035511 29989045

[19] Kumar S Jalan A Patowary BN. Laparoscopic appendectomy versus open appendectomy for acute appendicitis: a prospective comparative study. Kathmandu Univ Med J (KUMJ) 2016;14:244–48. 28814687

[20] Wilkinson E Aruparayil N Gnanaraj J. Barriers to training in laparoscopic surgery in low- and middle-income countries: a systematic review. Trop Doct 2021;51:408–14. 33847545 10.1177/0049475521998186 PMC8411480

[21] Kebede MA Tor DSG Aklilu T. Identifying critical gaps in research to advance global surgery by 2030: a systematic mapping review. BMC Health Serv Res 2023;23:946. 37667225 10.1186/s12913-023-09973-9 PMC10478287

